# Overexpression of CBS/H_2_S inhibits proliferation and metastasis of colon cancer cells through downregulation of CD44

**DOI:** 10.1186/s12935-022-02512-2

**Published:** 2022-02-16

**Authors:** Yuyang Zhang, Shanwen Chen, Jing Zhu, Shihao Guo, Taohua Yue, Hao Xu, Jianwen Hu, Zhihao Huang, Zeyang Chen, Pengyuan Wang, Yucun Liu

**Affiliations:** Department of General Surgery, Peking University First Hospital, Peking University, Beijing, 100034 China

**Keywords:** Colorectal cancer, Hydrogen sulfide (H_2_S), Cystathionine-beta-synthase (CBS), CD44, GYY4137

## Abstract

**Background:**

The role of hydrogen sulfide (H_2_S) in cancer biology is controversial, including colorectal cancer. The bell-shaped effect of H_2_S refers to pro-cancer action at lower doses and anti-cancer effect at higher concentrations. We hypothesized that overexpression of cystathionine-beta-synthase (CBS)/H_2_S exerts an inhibitory effect on colon cancer cell proliferation and metastasis.

**Methods:**

Cell proliferation was assessed by Cell Counting Kit-8 (CCK-8), clone-formation and sphere formation assay. Cell migration was evaluated by transwell migration assay. Intracellular H_2_S was detected by H_2_S probe. Chromatin immunoprecipitation (ChIP) analysis was carried out to examine DNA–protein interaction. Cell experiments also included western blotting, flow cytometry, immunohistochemistry (IHC) and immunofluorescence analysis. We further conducted in vivo experiments to confirm our conclusions.

**Results:**

Overexpression of CBS and exogenous H_2_S inhibited colon cancer cell proliferation and migration in vitro. In addition, overexpression of CBS attenuated tumor growth and liver metastasis in vivo. Furthermore, CD44 and the transcription factor SP-1 was probably involved in the inhibitory effect of CBS/H_2_S axis on colon cancer cells.

**Conclusions:**

Overexpression of CBS and exogenous provision of H_2_S inhibited colon cancer cell proliferation and migration both in vivo and in vitro. Molecular mechanisms might involve the participation of CD44 and the transcription factor SP-1.

**Supplementary Information:**

The online version contains supplementary material available at 10.1186/s12935-022-02512-2.

## Background

Colorectal cancer (CRC) is a life-threatening disease with the second-highest mortality of all human malignancies worldwide [1]. It is also the second-most common cancer in China, accounting for 12.2% of new cancer cases in 2020 [1]. However, the biological and molecular mechanisms of CRC are still elusive and liver metastases remain a major contributor of cancer related mortality in CRC.

Hydrogen sulfide (H_2_S), the third ‘gasotransmitter’ in addition to nitric oxide (NO) and carbon monoxide (CO), is synthesized mainly from L-cysteine through three pivotal enzymes: cystathionine-beta-synthase (CBS), cystathionine-gamma-lyase (CSE) and 3-mercapto-pyruvate sulfurtransferase (MPST) [2–4]. Endogenous H_2_S is widely involved in the delicate regulation of various physiological conditions, including nervous [5], cardiovascular [6], renal [7], gastrointestinal [8], reproductive [9] and respiratory [10] systems. Colon epithelium is constantly exposed to high levels of H_2_S derived from gut microbial metabolism [11]. Increased sulfide oxidation pathway, mainly composed of sulfide quinone oxidoreductase (SQR), thiosulfate-dithiol sulfurtransferase (TST) and ethylmalonic encephalopathy protein 1 (ETHE1), has been involved in detoxifying H_2_S in colon epithelial cells [11]. Previous studies including our work have validated the upregulation of CBS/H_2_S in CRC tumor specimens compared with patient-matched nonmalignant tissues [12]. Endogenous H_2_S was involved in the metabolic reprogramming and tumor associated angiogenesis in the CRC tumor microenvironment, contributing to the chemo-resistance phenotype. However, a plethora of studies have focused on the inhibitory effect of high levels of exogenous H_2_S on the proliferation and metastasis of cancer cells and several H_2_S donors have been developed. The bell-shaped model of the physiological role of H_2_S provided a convincing explanation to this paradox, which refers to pro-cancer action at lower doses of H_2_S and potential anti-cancer effect at higher levels [13]. Considering the dichotomous effect of H_2_S and the development of new era H_2_S donors, our study set out to investigate the declining part of the bell and hypothesize that overexpression of CBS/H_2_S might exhibit an anti-cancer activity on CRC cells.

CD44, a transmembrane glycoprotein and an important biomarker of cancer stem cells (CSCs) [14–16], is essential to many tumor cell activities, including proliferation and metastasis. CD44 has several variant isoforms generated through alternative mRNA splicing and these isoforms are reported to be associated with higher metastatic potential and poorer prognosis in various cancers, including CRC [17–21]. To our knowledge, there are no existing studies linking gasotransmitter H_2_S with cell surface molecule CD44 in any way.

Our results show that overexpression of CBS/H_2_S indeed exerts an inhibitory effect on the proliferation and migration of colon cancer cells both in vitro and in vivo. Mechanistically, CBS overexpression inhibits the expression of CD44, probably via attenuating the activation and nuclear translocation of the transcription factor SP-1.

## Methods

### Cell culture

HT-29, HCT-116 and Caco-2 cells were purchased from American tissue culture collection (ATCC, Manassas, VA, USA). Dulbecco’s Modified Eagle Medium (DMEM; Biological Industries, Israel) was used as the culture medium for HT-29 and Caco-2 cells while McCoy’s 5A Modified Medium (Biological Industries, Israel) was used for HCT-116, each supplemented with 10% fetal bovine serum (FBS) (Gibco, USA), 100 units/ml penicillin and 100 μg/ml streptomycin (Gibco, USA). All cells were maintained at 37 °C in a humidified atmosphere with 5% CO_2_.

### Gene editing

KO- (sh-CBS) was performed using lentiviruses with short hairpin RNA (shRNA) targeting CBS mRNA sequence (Sigma, USA). Lentiviruses with shRNA of scrambled sequence served as negative control (Sigma, USA). Caco-2 cells were infected at a MOI of 30 with 10 μg/ml of polybrene, and were then selected with 6 μg /ml puromycin for 7 days. Similarly, overexpression of CBS for HT-29 cell line was achieved through transfection with lentiviruses containing h-CBS sequence (Hanbio Biotechnology Co., Ltd., China) at a MOI of 30 with 10 μg/ml of polybrene followed by screening with 4 μg /ml puromycin for 7 days.

Stable knockout of CBS (KO-CBS) for HT-29 cell line was conducted using CRISPR/Cas9 system. Gene-specific sgRNA was designed to target CBS coding regions as shown below: CTGATGAGATCCTGCAGCAG. We first phosphorylated, annealed, and cloned the guide oligonucleotide into the BsmBI site of the pHBLV-U6-gRNA-EF1-CAS9-PURO vector (Hanbio Biotechnology Co., Ltd., China), and then verified the constructed vector by sequencing. Next, we transformed the transfer plasmid with the oligonucleotide into Escherichia coli strain DH5α bacteria, and used Plasmid DNA purification kit (Macherey–Nagel, Germany) to isolate the amplified plasmid from the bacteria. Transferable lenti-CAS-puro plasmid (Hanbio Biotechnology Co., Ltd., China), packaging plasmids psPAX2 (Hanbio Biotechnology Co., Ltd., China) and pMD2G (Hanbio Biotechnology Co., Ltd., China) were transfected into 293T cells to produce the lentivirus. Virus-containing supernatant was collected at 48 and 72 h post transfection and was used to infect Caco-2 cells. Sixteen hours after the infection, fresh medium containing 10 μg/ml puromycin was utilized to replace the lentivirus-containing medium. We collected the puromycin-resistant cells after 7 days of screening.

Finally, western blotting was used for all gene-edited cell lines to assess the silencing or overexpression efficiency.

### Cell counting kit-8 (CCK-8) assay

CCK-8 assay was performed to measure the capacity of cell proliferation using CCK-8 kit (Sigma-Aldrich, USA). Cells were seeded in 96-well plates at a density of 4000 cells per well with or without GYY4137 supplement. After incubation, 10 μl CCK8 were added to the wells at different time. The absorbance was measured at 450 nm by microplate reader (Bio-Rad, Hercules, CA, USA).

### Colony formation assay

Colony formation assay was used for cell proliferation analysis. Cells were seeded into 6-well plates at a density of 1000 cells per well for 14 days. Colonies were then fixed with 10% formaldehyde for 20 min and stained with 0.1% crystal violet for 10 min at room temperature. Colonies containing more than 50 cells were counted and the mean colony numbers were calculated. Each clone was plated in triplicate in each experiment.

### Sphere formation assay

Sphere formation assay was used to detect cell-renewal ability. Five hundred cells were seeded into 24-well non-treated cell culture plates (Nest, China). Serum- free F12/DMEM (Gibco, USA) supplemented with 2% B27 (Gibco, USA), 20 ng/ml human recombinant epidermal growth factor (PeproTech, USA), 20 ng/ml human recombinant fibroblast growth factor-10 (PeproTech, USA), 10 ng/ml human recombinant hepatocyte growth factor (PeproTech, USA), 100 units/ml antibiotics penicillin and 100 μg/ml streptomycin (Gibco, USA) were used for cell culture. After 10 days, spheres larger than 50 μm were counted and photographed under a light microscope. Each clone was plated in triplicate in each experiment.

### Transwell migration assay

For migration assay, 2.5 × 10^5^ cells suspended in 200 μl serum-free medium were added into the upper transwell chamber (8 mm, Corning Costar, USA), and 700 μl medium supplemented with 20% serum was placed in the lower chamber. Each transwell chamber contains a 6.5 mm diameter membrane with 8.0 μm pore size. After incubation, cells on the upper surface of membrane were removed gently with a cotton swab. Cells invading to the lower surface were fixed with methanol before staining with 0.1% crystal violet for 20 min at room temperature. The stained cells were counted in 5 randomly selected fields under a light microscope. Each clone was plated in triplicate in each experiment.

### Real-time quantitative PCR (RT-qPCR)

Total RNA from xenografts and cell lines were isolated using the TRIzol reagent (Invitrogen, Carlsbad, CA, USA) following the manufacturer’s instructions. Total RNA (1 mg) was eluted with RNase-free water and stored at – 80 °C. RT-qPCR was performed using SYBR-green PCR Master Mix in a Fast Real-time PCR 7500 System (Applied Biosystems). The primers for CD44 were as follows: 5′-CCTTTGATGGACCAATTACCATAAC-3′ (forward); 5′-TCAGGATTCGTTCTGTATTCTCCTT-3′ (reverse). GAPDH was used as the internal control. Fold change of CD44 was calculated by the 2^−ΔΔCt^ method.

### Western blotting

Total protein was extracted using RIPA buffer and the extracts containing equal quantities of protein (30 μg) were electrophoresed in 10% polyacrylamide gel, transferred with PVDF membranes and blocked for 1 h (5% BSA in TBS-Tween 20 buffer) at room temperature. Incubations with primary antibodies to detect CD44, CBS, and β-actin (CST, USA) were followed by incubations with secondary antibodies conjugated with horseradish peroxidase (CST, USA). Blots were developed with ECL detection reagents (Millipore, USA). Images were collected utilizing Syngene GeneGenius gel imaging system (Syngene, UK) according to the manufacturer's instructions.

### Immunohistochemistry (IHC) analysis

Xenografts from subcutaneous injection and liver samples were immediately fixed in 10% neutral buffered paraformaldehyde. After fixation, the tissue was dehydrated in a graded ethanol series and then embedded in paraffin. Each section (3-μm) of the paraffin-embedded tissue was mounted on a glass slide and either stained with hematoxylin and eosin (H&E) or processed for IHC. For the latter, each slide was completely deparaffinized by immersion in xylene twice for 10 min and rehydrated with water following incubation in graded ethanol (100, 90, 80, and 70%). The antigen retrieval procedure was carried out by microwaving the slides for 10 min in citrate buffer (pH 6.0; Biogenex, San Ramon, CA) followed by incubation in 3% H_2_O_2_ in methanol to block endogenous tissue peroxidase activity. The sections were blocked with 1.5% goat serum for 1 h and incubated with CD44 or Ki-67 antibody overnight at 4 °C. Mouse anti-CD44 (CST, USA) and mouse anti-Ki-67 (CST, USA) antibody was used. After washing with PBS, slides were then incubated with a biotinylated secondary antibody for 30 min at room temperature. The antigen signal was amplified using the ABC method (Vectastain ABC kit, catalogue no. PK-6105, Vector Laboratories, Burlingame, CA). The antigen–antibody–avidin complex was detected using the chromogenic substrate 3,3-diaminobenzidine, which produced a dark brown color. Immunostained sections were counterstained with hematoxylin and examined by light microscopy. H-score was used for the semi-quantitive analysis of IHC results. The calculation of H-score was based on the intensity of staining (3, strong; 2, moderate; 1, weak; 0, none) and the proportions of positively stained tumour cells as previously described (H-score = % strong staining × 3 + % moderate staining × 2 + % weak staining × 1 + % no staining × 0) [22]. For each sample, five fields were randomly selected and the average H-score was calculated.

### Immunofluorescence assay

For the immunofluorescence assay, after fixation with 4% paraformaldehyde for 10 min, PBS was used to gently wash the cells thrice. Then, the cells were immunostained with primary antibodies targeting SP-1 (CST, USA) at 4 °C overnight, and the secondary antibodies used were Alexa Fluor 488 donkey anti-rabbit IgG (Thermo Fisher Scientific, USA). Lastly, the cells were again washed with PBS and then mounted with ProLong Gold mounting medium with DAPI (Molecular Probes, USA). The sections were observed by confocal laser scanning microscopy (Zeiss LSM780, Carl Zeiss, Germany). Pearson’s correlation coefficient was used for the semi-quantitive analysis of the colocalization values as previously described [23].

### Fluorescent detection of intracellular H_2_S

To visualize intracellular H_2_S level, 2 × 10^4^ cells were seeded in a glass-bottom 35 mm well (Corning, USA) and cultured overnight. After adding 10 µmol/L of H_2_S-specific near-infrared fluorescence probe to the culture medium and incubated for 30 min, the living cells were immediately sent for fluorescence imaging [24].

### Flow cytometry

For surface marker detection, the cells were collected and resuspended at a density of 1 × 10^4^ per test. After incubation for 30 min at room temperature with CD44-APC antibody (eBioscience, USA), the cells were washed twice with PBS and resuspended in 400 µL of PBS for flow cytometry using Calibur 2 (Beckman Coulter, USA). The results were analyzed with FlowJo software (Tree Star, Ashland, OR, USA). The mean fluorescence intensity (MFI) ratio (sample ΔMFI (specific marker MFI − isotype control MFI)/control sample ΔMFI × 100) was calculated for all samples.

### Chromatin immunoprecipitation (ChIP) assay

ChIP assays were performed according to the instructions of Agarose ChIP Kit (Thermo Fisher Scientific, USA). Antibody against SP-1 (CST, USA) was used to precipitate the DNA–protein complex and subsequently elute the DNA from the antibody. Primers specific for the CD44 promoter were 5′- CTCTTTCCACTTGGAAGATTCACCA-3′ (forward) and 5′- TGGATATCCTGGGAGAGGAGCT-3′ (reverse). The immunoprecipitated DNA was amplified by real-time PCR using SYBR-green PCR Master Mix in a Fast Real-time PCR 7500 System (Applied Biosystems).

### Xenograft model

All animal experiments were approved by the Institutional Animal Care and Use Committee (IACUC) of Peking University First Hospital. Twelve male BALB/c nude mice at age of 6 weeks were purchased from Beijing Vital River Laboratory Animal Technology Co., Ltd.. Each mouse was subcutaneously inoculated with 1 × 10^6^ of HT-29 cells in a volume of 200 μl PBS with or without CBS overexpression. After a palpable tumor was developed, the tumor length A and width B were measured twice a week using a caliper. The formula used for calculating the tumor volume was A × B2/2. Mice were humanely sacrificed by exposure to a fixed flow rate of CO_2_ (30% chamber replacement rate) 34 days after inoculation. Subcutaneous tumor grafts were harvested and analyzed by western blotting and IHC analysis.

### Intrasplenic injection model

Twelve 6-week-old male Balb/c nude mice were purchased from Beijing Vital River Laboratory Animal Technology Co., Ltd.. To investigate the tumor metastasis in vivo, 1 × 10^6^ of HT-29 cells containing stably-expressed human CBS sequence or empty vector in a volume of 100 μl in PBS were injected into spleen subcapsular followed by spleen-resection after 5 min. After 8 weeks, the livers of nude mice were surgically removed after euthanasia with CO_2_ (30% chamber replacement rate), fixed in 10% neutral buffered formalin, embedded in paraffin, and prepared into 3-μm sections for H&E staining and IHC analysis.

### Statistical analysis

Data are expressed as means ± SD. All statistical analyses were undertaken using Prism for macOS software, version 8.4.1 (GraphPad Software, La Jolla, CA, USA). Student’s t-test or the Mann–Whitney U-test were used for comparisons between two experimental groups with or without normal distribution, respectively. p-values < 0.05 were considered statistically significant.

## Results

### Overexpression of CBS inhibits HT-29 cell proliferation, clone formation, sphere formation, and migration

**Fig. 1:**
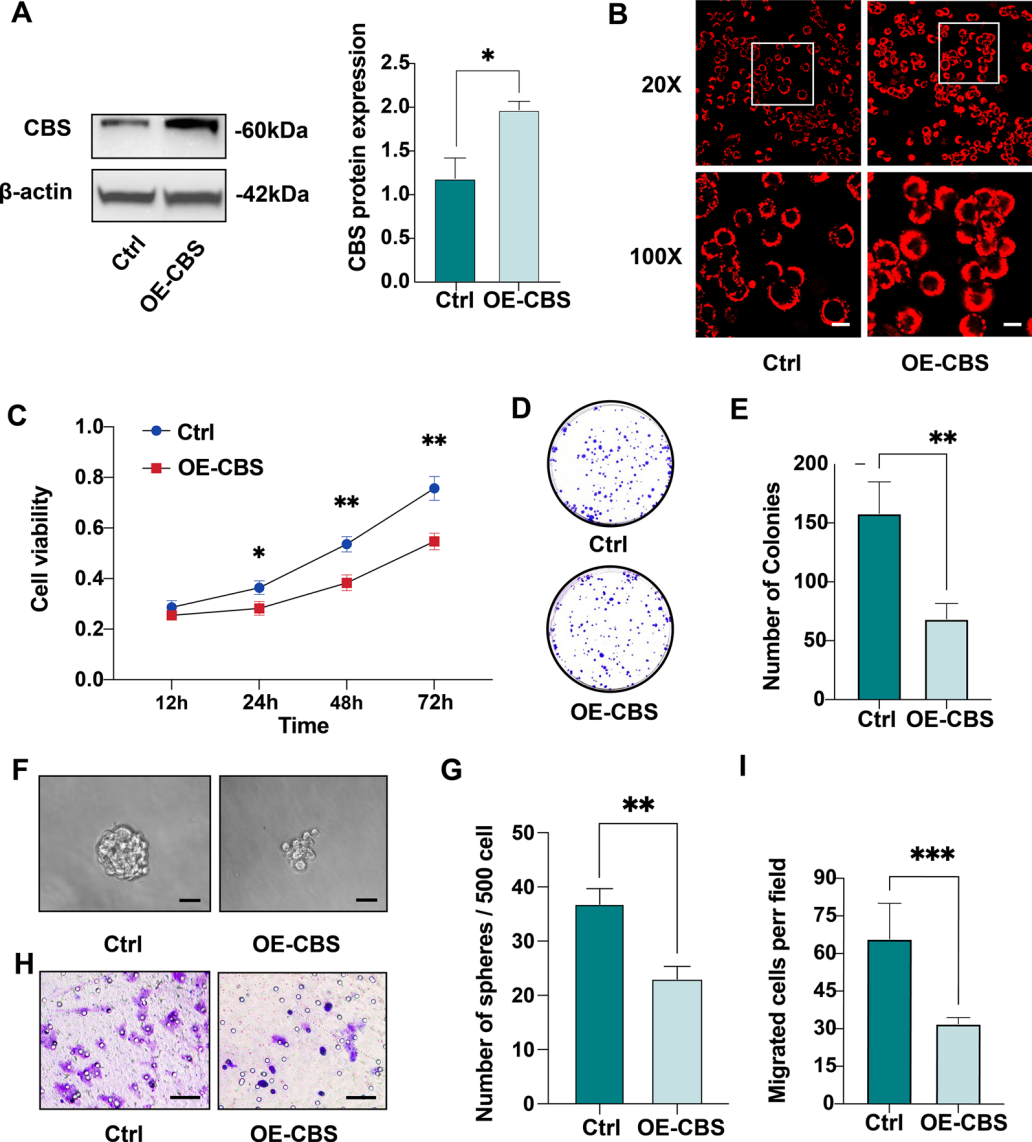
Overexpression of CBS inhibits HT-29 cell proliferation, clone formation and migration. **A** Overexpression of CBS protein in HT-29 cells after lentivirus infection (*p < 0.05). **B** H_2_S fluorescent probe visualizing the intracellular levels of H_2_S in HT-29 control and CBS-overexpressing cells. Scale bar, 20 μm. **C** The proliferation of HT-29 control and CBS-overexpressing cells assessed by CCK-8 assay (*p < 0.05, **p < 0.01). **D**, **E** Colony-formation assay of HT-29 control and CBS-overexpressing cells (**p < 0.01). **F**, **G** Sphere formation assay of HT-29 control and CBS-overexpressing cells (**p < 0.01). Scale bar, 50 μm. **H**, **I** Migration capacity determined by transwell assay in HT-29 control and CBS-overexpressing cells (***p < 0.001). Scale bar, 100 μm

In colorectal tumor tissue and cancer-derived cell lines, the expression of CBS is upregulated and closely related to tumor growth and carcinogenesis. At the same time, the dual-effect of several gasotransmitters, including H_2_S, CO and NO, has been reported. To investigate the biological function as well as therapeutic potentials of CBS/H_2_S axis, HT-29 cell line with stable overexpression of CBS was generated. The approximately twofold upregulation of CBS was confirmed by western blotting, and the increased production of H_2_S was verified by a fluorescent H_2_S probe through fluorescence analysis (Fig. 1A, B). To examine whether overexpression of CBS affects CRC cell proliferation, we performed a CCK-8 assay and the results showed that overexpression of CBS significantly inhibited HT-29 proliferation from 24 to 72 h (vs. control vector, *p < 0.05, **p < 0.01; Fig. 1C). Consistent with the cell viability analysis, a colony formation assay indicated that overexpression of CBS significantly reduced the number of HT-29 cell clones (**p < 0.01; Fig. 1D, E). Next, to further examine the effect of CBS overexpression on cell-renewal capacity, we conducted a sphere formation assay. As expected, CBS-overexpressing HT-29 cells formed smaller and less spheres than that of control cells (**p < 0.01; Fig. 1F, G). Finally, we explored whether CBS overexpression affected the migration of HT-29 cells using 24-h transwell assays. We observed that cell migration was significantly attenuated in CBS-overexpressing HT-29 cells (***p < 0.001; Fig. 1H, I). In general, these results suggest that CBS overexpression inhibits CRC cell proliferation, clone formation, sphere formation, and migration in vitro.

### Exogenous H_2_S inhibits CRC cell proliferation and migration.

**Fig. 2:**
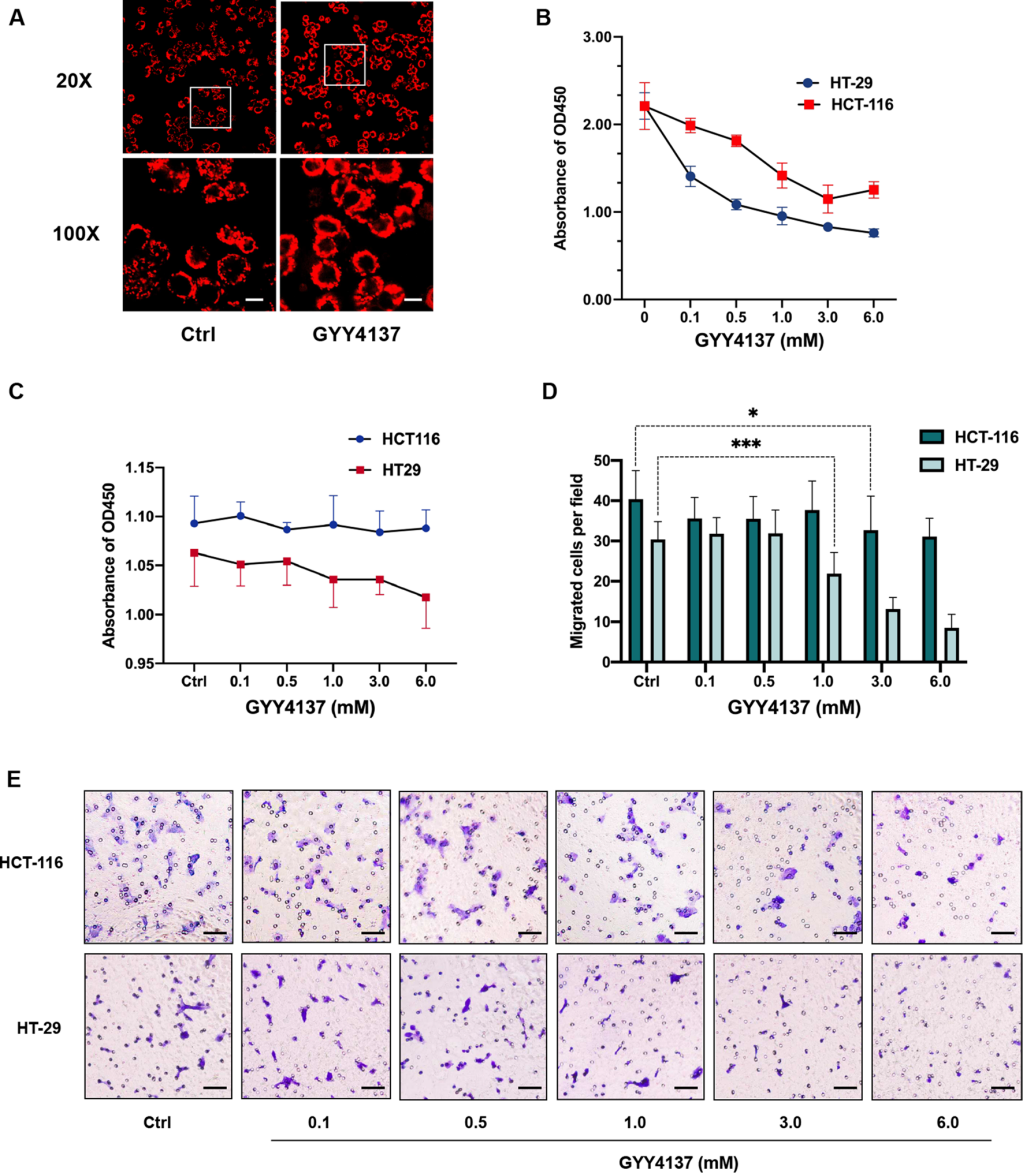
Exogenous H_2_S inhibits CRC cell proliferation and migration. **A** GYY4137 increases the intracellular levels of H_2_S in HT-29 cells visualized by H_2_S fluorescent probe. Scale bar, 20 μm. **B** GYY4137 inhibits the proliferation of HT-29 and HCT-116 cells assessed by CKK-8 assay (*p < 0.05, ***p < 0.001). **C** Proliferation capacity remains unimpaired after 18-h of GYY4137 treatment in HT-29 and HCT-116 cells. **D**, **E** Migration capacity determined by 18-h transwell assay in HT-29 and HCT-116 cells treated with GYY4137 (**p < 0.01, ***p < 0.001). Scale bar, 100 μm

To further determine the inhibitory effect of CBS overexpression and the resulting increased rate of H_2_S production on CRC cells, we treated both HCT-116 and HT-29 cells with a slow-releasing H_2_S donor, GYY4137. We first visualized the elevated intracellular H_2_S concentration with the fluorescent H_2_S probe (Fig. 2A). Then, we investigated the proliferation ability of CRC cells using CCK-8 assay. Consistent with the CBS overexpressing results, GYY4137 treatment inhibited the growth of HCT-116 (1.0 ~ 6.0 mM) and HT-29 cells (0.1 ~ 6.0 mM) in a dose-dependent manner (vs. 0 mM GYY4137, *p < 0.05, ***p < 0.001; Fig. 2B). In addition, results from 18-h CCK-8 and transwell assay demonstrated that exogenous H_2_S from GYY4137 attenuated the migration capacity of HCT-116 (1.0 ~ 6.0 mM) and HT-29 cells (0.1 ~ 6.0 mM) dose-dependently (**p < 0.01, ***p < 0.001; Fig. 2C–E). Taken together, our results show that CBS overexpression and the consequent over-production of H_2_S lead to an impaired proliferation and migration ability of CRC cells.

### Overexpression of CBS attenuates CRC cell growth and liver metastasis in vivo.

**Fig. 3:**
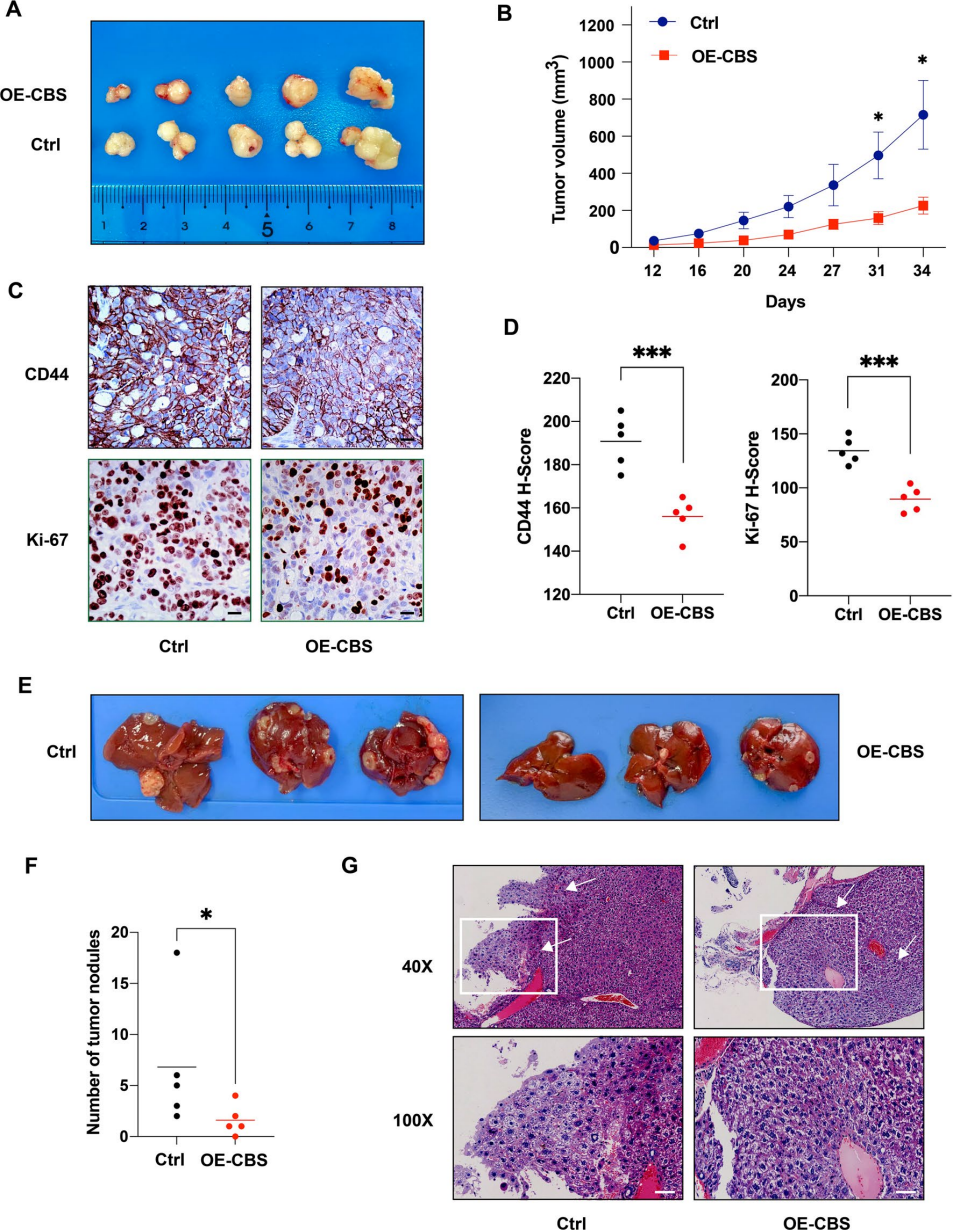
Overexpression of CBS attenuates CRC cell growth and liver metastasis in vivo. **A** Typical macroscopic pictures of xenograft tumors from subcutaneous injection in HT-29 control and CBS-overexpressing group (n = 6). **B** Tumor growth trend in HT-29 control and CBS-overexpressing group (*p < 0.05). **C** Immunohistochemistry results of subcutaneous xenografts from HT-29 control and CBS-overexpressing group. Scale bar, 50 μm. **D** Statistical analysis of immunohistochemistry results of subcutaneous xenograts from HT-29 control and CBS-overexpressing group based on H-score. (***p < 0.001). **E** Typical macroscopic pictures of liver specimens harvested at 8 weeks after intrasplenic injection in HT-29 control and CBS-overexpressing group (n = 6). **F** Statistical analysis of macroscopic metastatic tumor nodules on the liver surface tissues in HT-29 control and CBS-overexpressing group (*p < 0.05). **G** Typical microscopic pictures of liver metastatic areas in HT-29 control and CBS-overexpressing group. White arrows indicate metastatic areas. Scale bar, 50 μm

To clarify the in vivo effect of CBS overexpression on CRC cell proliferation, we established xenograft mouse model by injecting HT-29 cells with stable CBS overexpression or control cells into the left dorsal flank of nude mice (n = 6 for each group). The results indicated that CBS overexpression inhibited tumor growth rate and the difference was significant on day 31 and 34 (*p < 0.05; Fig. 3A, B). We also performed IHC staining of harvested xenografts and the results showed that CD44 and Ki-67 expression level were significantly higher in the control group, which was quantified by H-Score (***p < 0.0001; Fig. 3C, D). Next, we assessed the effect of CBS overexpression on tumor metastasis by intrasplenic injection of HT-29 cells into nude mice (n = 6 for each group). Two out of 12 mice developed evident subcutaneous masses and was therefore excluded from further analysis. Eight weeks after injection, the CBS-overexpressing group developed significantly fewer and smaller liver metastasis nodules in gross morphology (*p < 0.05; Fig. 3E, F). Liver metastases from both groups were then confirmed by H&E staining and typical fields under light microscope were demonstrated (Fig. 3G). Together, these results demonstrate that CBS overexpression attenuates CRC cell growth and liver metastasis in vivo.

### CD44 and the transcription factor SP-1 is involved in the inhibitory effect of CBS/H_2_S axis on CRC cells

**Fig. 4:**
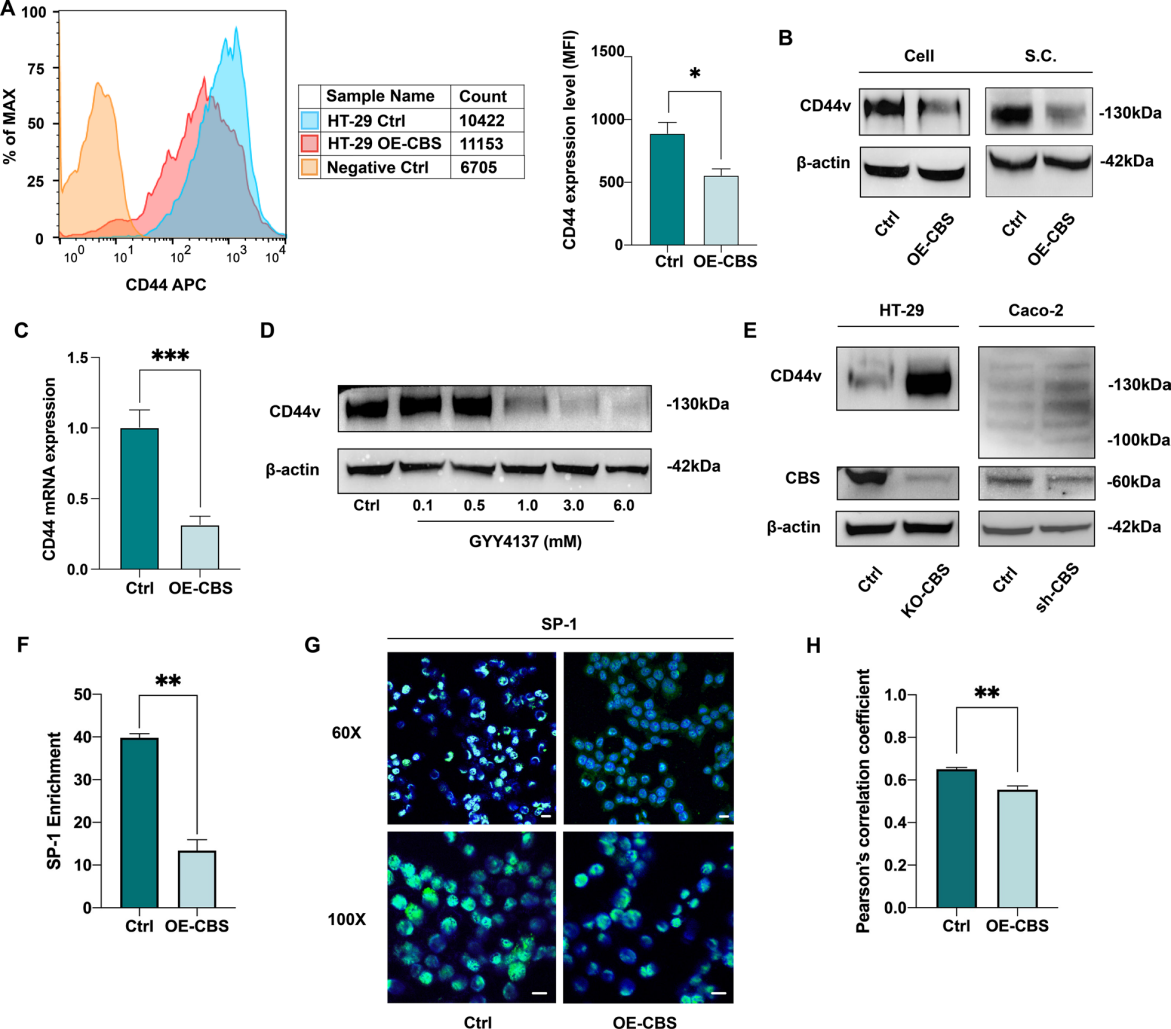
CD44 and the transcription factor SP-1 is involved in the inhibitory effect of CBS/H_2_S axis on CRC cells. **A** CD44 expression level detected by flow cytometry and quantified through MFI in HT-29 cell line. (*p < 0.05). **B** Decreased CD44v protein expression after CBS overexpression in HT-29 cell line as well as xenograft samples. **C** Decreased CD44v mRNA expression after CBS overexpression in HT-29 cell line as well as xenograft samples (***p < 0.001). **D** Dose-dependent decrease in CD44v protein expression upon GYY4137 supplement. **E** Elevated CD44v protein expression in HT-29 KO-CBS cell line and Caco-2 sh-CBS cell line. **F** ChIP assay and following RT-qPCR results showing different level of SP-1 enrichment within the CD44 promoter region in HT-29 control and CBS-overexpressing cells (**p < 0.01). Fold enrichment relative to IgG. **G** Immunofluorescence assay of SP-1 and DAPI in HT-29 control and CBS-overexpressing cells, depicting SP-1 intracellular distribution. Scale bar, 20 μm. **H** Analysis of colocalization of SP-1 and DAPI using Pearson's correlation coefficient (**p < 0.01)

CD44 is a large family of transmembrane glycoproteins well known for its pivotal role in regulating cell proliferation, migration, invasion, and stemness. To explore how CBS/H2S axis exerts its inhibitory effect on CRC cells, we examined the effect of CBS overexpression on stemness marker CD44. Flow cytometry analysis showed that, the MFI ratio of CD44 was significantly higher in the control group comparing to the CBS overexpressing group (Fig. 4A). Next, we determined the association between CBS overexpression and CD44 expression through RT-qPCR and western blotting in HT-29 cell lines and xenografts from nude mice model. The results suggested that CBS overexpression led to a significant reduction in CD44 variant (CD44v) expression on both mRNA and protein levels (Fig. 4B, C).

We then verified our findings with exogenous H_2_S donation from GYY4137 on HT-29 cell line. When given at a higher concentration (1.0 ~ 6.0 mM), a significant decrease in CD44v expression was observed in a dose-dependent manner (Fig. 4D). To further investigate whether the inhibitory effect of CBS/H_2_S on CD44v expression was dependent on a relatively high CBS level, we established a sh-CBS Caco-2 cell line by shRNA lentivirus system and a KO-CBS HT-29 cell line by CRISPR/Cas9 technology (Fig. 4E). Western blotting results clearly demonstrated that CBS downregulation promoted CD44v expression (Fig. 4E).

Then, we set out to investigate the mechanism underlying the inhibitory effect of CBS/H_2_S on CD44v levels. It has been reported that the CD44 gene promoter region contains SP-1 binding sites [25], and that SP-1 phosphorylation level is associated with H_2_S and p38/MAPK pathways [26]. Therefore, we hypothesized that the participation of CD44v in the regulation of CBS/H_2_S axis on CRC cells was mediated through RNA binding protein, SP-1. We conducted ChIP assays followed by quantitative PCR with primers specifically targeting the SP-1 binding region in the promoter sequence of human CD44. The ChIP results showed that overexpression of CBS led to a significant decrease in SP-1 recruitment to the CD44 promoter (Fig. 4F). We then carried out an immunofluorescence assay to further determine the cellular distribution of SP-1. Semi-quantitive analysis showed a significant reduction in the colocalization of SP-1 and DAPI in CBS-overexpressing cells determined by Pearson's correlation coefficient, indicating a decreased nuclear enrichment of SP-1 in HT-29 cells with CBS overexpression (**p < 0.01, Fig. 4G, H).

## Discussion

The potential role of H_2_S in colorectal cancer has been a subject of extensive studies. Controversy still exists concerning whether this rather new gasotransmitter exerts a pro-cancer or anti-cancer effect. IHC and western blotting analysis using human colon cancer specimens revealed a distinct increased expression of CBS in tumor tissues compared with adjacent normal mucosa slices. Contrary to the result at protein level from limited patient samples, no such difference could be detected on mRNA levels based on the analysis of GEPIA2 database, which contains global gene expression data from TCGA and GTEx (Additional file 1: Fig S1; http://gepia2.cancer-pku.cn/#index). Although previous studies have mainly focused on the pro-tumor aspect of CBS/H_2_S, including maintaining cellular bioenergetics, promoting tumorigenesis, and stimulating angiogenesis and vasorelaxation [27], evidence from both in vitro and in vivo experiments supporting the anti-cancer effect of H_2_S are also accumulating [28].

To decipher the bewildering biological and pharmaceutical role of CBS/H_2_S axis, we conducted several in vitro and in vivo experiments, mainly focusing on cell lines with high CBS expression (HT-29 and HCT-116) [12]. The current study indicated that overexpression of CBS inhibited CRC cell proliferation, clone formation, sphere formation and migration. Consistent with the in vitro results, attenuated xenograft growth rate and reduced liver metastasis was observed in cells with CBS overexpression in nude mice model. We then identified the involvement of CD44 in the effect of CBS/H_2_S axis on CRC cells, which is a well-known transmembrane marker for its pro-cancer and stemness-maintenance function. It is worth noticing that the predominant form of CD44 examined in HT-29 cell line by western blotting was CD44v. Certain CD44 isoforms, such as CD44v6, are suggested to possess cancer-initiating ability [29]. CD44v was derived from mRNA alternative splicing and they were reported to have a positive correlation with the degree of tumor aggressiveness, especially the characteristics related to tumor metastasis [17–21]^.^

After verification of the CD44-mediated regulation pattern, we further explored the molecule mechanism underlying the regulatory effect of CBS/H_2_S on the expression of CD44. H_2_S has been reported to inhibit the phosphorylation process of a transcription factor, SP-1, and thus suppress its functional activity [26]. Moreover, SP-1 could bind to the promoter region of CD44 [25]. Thus, we conducted further experiments focusing on the function and nuclear translocation of SP-1. In line with our hypothesis, the results from ChIP assays indicated an attenuated interaction between CD44 mRNA and SP-1 in CBS-overexpressing HT-29 cell line. Finally, we discovered that overexpression of CBS inhibited nuclear enrichment of SP-1 through immunofluorescence staining and Pearson's correlation analysis. Taken together, our study suggested a SP-1/CD44-mediated inhibitory effect of CBS/H_2_S axis on the proliferation, migration and metastasis capacity of CRC cells.

The seemingly unorthodox results from our study could partly be explained by the dual-effect of H2S. Indeed, it has been reported that H_2_S, along with NO and CO, exhibited a pro-cancer effect at rather low concentrations and an anti-cancer action at higher concentrations [30]. Moreover, the tumor-suppressing role of different H_2_S donors has been preliminarily tested both in vitro and in vivo. Lee et al. first reported the anti-cancer effect of the slow-releasing H_2_S donor, GYY4137, by promoting cell cycle arrest and apoptosis [31]. Faris et al. discovered that exogenous administration of H_2_S suppresses proliferation in primary cultures of metastatic CRC cells by inducing an increase in intracellular flux of Ca^2+^ [32]. Modis et al. found that an allosteric CBS activator, S-adenosyl-l-methionine (SAM), inhibited HCT-116 cell proliferation and bioenergetics at higher levels (3 mM) or after longer-term exposure (72 h), although the inhibitory effect appeared to result from CBS-independent pharmacological mechanisms [33]. Other potential mechanisms underlying the anti-cancer effect of H_2_S administration including uncontrolled cellular acidification and inhibition of cell survival signaling pathways [13].

As for the underlying mechanism, the participation of SP-1/CD44 in the regulation of CBS/H_2_S on CRC cells should be interpreted rather cautiously, bearing in mind the complexity of the signaling pathway and cascades triggered by the fluctuation in CBS expression and H_2_S level in different tissues. Our results showed that overexpression of CBS attenuated the recruitment of SP-1 to the promoter region of CD44 mRNA, suggesting that CBS/H_2_S potentially modulates CD44 expression via the transcription factor SP-1. Indeed, Wu et al. reported that overexpression of CSE, another important H_2_S synthase, significantly suppressed SP-1, p38 and ERK1/2 activation in rheumatoid arthritis, and that SP-1 activation was inhibited by p38 and ERK [26]. These data suggested that H_2_S might negatively regulate SP-1 activation through inhibition of MAPK pathway. H_2_S modulated protein activity mainly through two ways: protein sulfhydration or intracellular formation of polysulfides followed by oxidative inactivation of proteins [34]. The exact mechanisms of how CBS/H_2_S axis controls CD44 expression via SP-1, either via protein sulfhydration or oxidative stress, remain to be further explored.

Based on the results from our study as well as many others, we hypothesized that, at a relatively low expression level, the upregulation of CBS/H_2_S triggers a series of downstream reactions that promote the growth and dissemination of CRC cells, which override the tumor-suppressing effect stemming from the downregulation of CD44. When the CBS/H_2_S axis is already highly-activated, however, a further upregulation of CBS or exogenous donation of H_2_S would lead to a major decrease in CD44 expression. Under such circumstances, CBS overexpression and exogenous H_2_S manifests tumor-suppressing activities in CRC cells, including inhibition of proliferation, decreased clone formation, attenuation of migration, as well as an impaired capacity of in vivo xenograft growth and liver metastasis. Apart from colon cancer, the anti-tumor activity of H_2_S has been widely discovered in various cancer cell types, including gastric, hepatic, breast and melanoma cancer cells [35]. Although clinical trials investigating H_2_S donor for cancer therapy have not yet been conducted, given the context-dependent role of CBS/H_2_S in tumor development, the therapeutic potential of H_2_S supplement in a subgroup of patients with high levels of CBS expression merits additional study.

## Conclusions

In conclusion, our study indicated that endogenous overexpression of CBS as well as exogenous H_2_S could inhibit CRC cell proliferation and migration both in vivo and in vitro. Moreover, the anti-tumor activity of CBS/H_2_S axis was largely attributed to the inhibition of a pivotal stem cell marker, CD44. CBS/H_2_S negatively modulated CD44 expression through the transcription factor SP-1. Further researches are still warranted to delineate the molecular mechanisms and therapeutic potential of CBS/H_2_S in CRC.

## Supplementary Information

**Additional file 1: Figure S1.** GEPIA2 database depicting CBS mRNA expression levels in normal and tumor tissues of COAD and READ patients.

## Data Availability

The datasets generated and/or analysed during the current study are available in the GEPIA2 database repository, http://gepia2.cancer-pku.cn/#index. Additionally, the data are available to interested researchers from the corresponding author on reasonable request.

## Abbreviations

H_2_S: Hydrogen sulfide
CCK-8: Cell counting kit-8
ChIP: Chromatin immunoprecipitation
IHC: Immunohistochemistry
CBS: Cystathionine-beta-synthase
CRC: Colorectal cancer
NO: Nitric oxide
CO: Carbon monoxide
CSE: Cystathionine-gamma-lyase
MPST: 3-Mercapto-pyruvate sulfurtransferase
SQR: Sulfide quinone oxidoreductase
TST: Thiosulfate-dithiol sulfurtransferase
ETHE1: Ethylmalonic encephalopathy protein 1
CSCs: Cancer stem cells
ATCC: American tissue culture collection
DMEM: Dulbecco’s Modified Eagle Medium
shRNA: Short hairpin RNA
sh-CBS: CBS knockdown with shRNA targeting CBS mRNA sequence
KO-CBS: CBS knockout with CRISPR/Cas9 system
H&E: Hematoxylin and eosin
MFI: Mean fluorescence intensity
IACUC: Institutional Animal Care and Use Committee
CD44v: CD44 variant
SAM: S-adenosyl-l-methionine
