# Supplementary figures and images for: Overexpression of CBS/H2S inhibits proliferation and metastasis of colon cancer cells through downregulation of CD44
Source: Cancer Cell Int. 2022 Feb 16;22:85. doi: 10.1186/s12935-022-02512-2 (PMC8848668; doi:10.1186/s12935-022-02512-2)

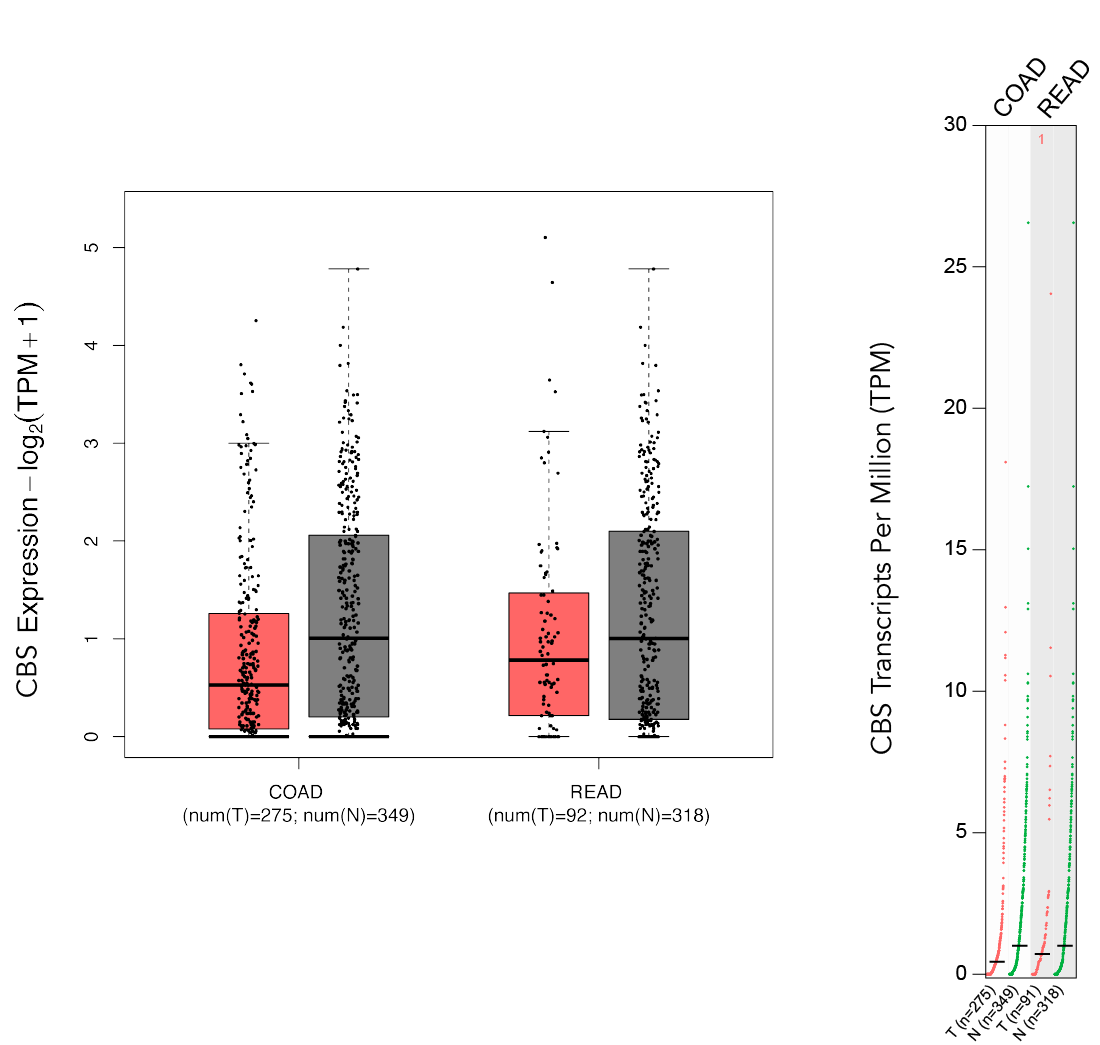

Supplement: Supplementary file 1 — Additional file 1: Figure S1. GEPIA2 database depicting CBS mRNA expression levels in normal and tumor tissues of COAD and READ patients. [file 12935_2022_2512_MOESM1_ESM.png]
